# Independent risk factors for an increased incidence of thromboembolism after lung transplantation

**DOI:** 10.1007/s11239-022-02748-9

**Published:** 2022-12-10

**Authors:** Isabelle Moneke, Ecem Deniz Ogutur, Johannes Kalbhenn, Ina Hettich, Bernward Passlick, Wolfgang Jungraithmayr, Omer Senbaklavaci

**Affiliations:** 1 Department of Thoracic Surgery, Faculty of Medicine, Medical Center, University of Freiburg, Hugstetter Str. 55, 79106 Freiburg, Germany; 2 Department of Anaesthesiology and Intensive Care Medicine, Faculty of Medicine, Medical Center, University of Freiburg, Freiburg, Germany; 3 Department of Pneumology, Faculty of Medicine, Medical Center, University of Freiburg, Freiburg, Germany

**Keywords:** Thromboembolism, Pulmonary embolism, Stroke, Metabolic syndrome, Lung transplantation

## Abstract

**Background:**

Thromboembolism (TE) after lung transplantation (LTX) is associated with increased morbidity and mortality. The aim of this study is to analyze the incidence and outcome of venous and arterial thromboembolic complications and to identify independent risk factors.

**Patients and methods:**

We retrospectively analyzed the medical records of 221 patients who underwent LTX at our institution between 2002 and 2021. Statistical analysis was performed using SPSS and GraphPad software.

**Results:**

74 LTX recipients (33%) developed TE. The 30-days incidence and 12-months incidence were 12% and 23%, respectively. Nearly half of the patients (48%) developed pulmonary embolism, 10% ischemic stroke. Arterial hypertension (p = 0.006), a body mass index (BMI) > 30 (p = 0.006) and diabetes mellitus (p = 0.041) were independent predictors for TE. Moreover, a BMI of > 25 at the time of transplantation was associated with an increased risk for TE (43% vs. 32%, p = 0.035). At the time of LTX, 65% of the patients were older than 55 years. An age > 55 years also correlated with the incidence of TE (p = 0.037) and these patients had reduced overall post-transplant survival when the event occurred within the first postoperative year (59% vs. 72%, p = 0.028).

**Conclusions:**

The incidence of TE after LTX is high, especially in lung transplant recipients with a BMI > 25 and an age > 55 years as well as cardiovascular risk factors closely associated with the metabolic syndrome. As these patients comprise a growing recipient fraction, intensified research should focus on the risks and benefits of regular screening or a prolonged TE prophylaxis in these patients.

*Trial registration* number DKRS: 00021501.

**Supplementary Information:**

The online version contains supplementary material available at 10.1007/s11239-022-02748-9.

## Highlights

- The incidence of arterial and venous thromboembolism after lung transplantation is high, notably in recipients with a BMI > 25 and an age > 55 years.
- Moreover, cardiovascular risk factors closely associated with the metabolic syndrome are independent risk factors for venous thromboembolism.
- Intensified research should focus on the risks and benefits of regular screening or a prolonged thromboembolism prophylaxis in patients at risk, especially in the first postoperative year.

## Introduction

Lung transplantation is the most efficient treatment option for selected patients with end stage chronic lung disease [1], such as idiopathic pulmonary fibrosis or chronic obstructive pulmonary disease (COPD) [1]. Although the median survival rates have improved over the last decade, chronic lung allograft dysfunction (CLAD)—the hallmark of chronic lung allograft rejection—is responsible for a 5-years survival of only 55% [2]. It occurs in about half of the patients within 5 years after transplantation and up to now, there is no effective treatment available.

Since the introduction of the Lung Allocation Score (LAS) in 2005, the recipient population has changed, and older patients with age-related comorbidities are now more likely to receive an organ [3]. According to the 2013 report of the Registry of the International Society for Heart and Lung Transplantation, the median age at the time of LTX has gradually increased from 45 to 55 years over the preceding decade [4]. This implies the need to adjust to a growing fraction of patients of more than 60 or even 65 years of age with a higher risk of morbidity and mortality after lung transplantation [5].

Aside from postoperative infections, lung transplant recipients may experience cardiovascular complications, such as thromboembolism [6–8]. Thromboembolism (TE) is a well-known complication after surgery and the level of risk mainly depends on the surgery performed [9]. Acute pulmonary embolism is the most dangerous form of venous TE and can be fatal if left untreated [10]. It is associated with cardiac arrhythmia and right ventricular dysfunction and has an overall mortality rate of up to 10% [10, 11]. The reported incidence of thromboembolic complications after LTX is considered higher compared to other cardiothoracic surgeries [12] but varies widely between studies (6–44%) [6, 8, 13–15]. In our study, as in most of the others, only clinical symptoms prompted further investigation, so thromboembolic events might have been missed. This is supported by studies that implemented a regular screening protocol, where a higher incidence of TE was reported compared to studies without such a protocol [12]. Solid organ transplantation itself is recognized as an independent risk factor for thromboembolic events [16]. There are several underlying factors promoting TE: the surgical trauma itself induces inflammation leading to a prothrombotic state, immobilisation und fluid imbalance results in decreased venous flow. Side effects from immunosuppressive medication such as calcineurin inhibitors or corticosteroids, which impair glucose tolerance and induce post-transplant diabetes [17] further enhance the risk to develop thrombosis. Moreover, bacterial and viral infections have been shown to increase the risk for thrombotic events [9, 12, 18]. With increasing age, a growing number of patients develop traits of the metabolic syndrome, a heterogeneous clinical entity which includes the co-occurrence of overweight, impaired glucose tolerance, dyslipidaemia and hypertension leading to cardiovascular disease and diabetes mellitus [19, 20]. Two additional components underlined by the conference paper on the definition of the metabolic syndrome of the American Heart Association are a proinflammatory and a prothrombotic state [21]. A growing understanding is that venous TE is a chronic process which shares similar risk factors and pathophysiology, e.g., endothelial dysfunction, with atherothrombosis and coronary artery disease [9, 22]. Thus, a higher age at the time of transplantation, often accompanied by metabolic and cardiovascular comorbidity, makes a growing fraction of the lung transplant recipients particularly vulnerable to thromboembolic complications.

Clinical trials in general -, urologic -, and orthopaedic surgery have shown that the incidence of venous postoperative TE can be significantly reduced by interventions like early mobilization and adequate pharmacologic thrombosis prophylaxis to a range of 1.1–10.6% [9, 23]. While TE in general is associated with increased morbidity and hospital length of stay as well as reduced overall survival [24], there is limited data regarding the optimal management of these patients.

The aim of this study is to analyse the incidence of arterial and venous thromboembolic events in our lung transplanted patient cohort and identify independent risk factors. Furthermore, we make an attempt to discuss a regular screening during follow-up for patients at risk and the potential need for a personalised prophylaxis regime after surgery for a growing number of our patients.

## Methods

### Design and study population

We performed a retrospective single centre analysis of patients who underwent LTX at the Department of Thoracic Surgery, Medical Centre—University of Freiburg between March 2003 and June 2021. A total of 221 patients were identified (115 males and 106 females). Patients with combined transplantations, such as heart–lung transplantations, were excluded.

All patients underwent regular clinical follow-ups, including bronchoscopy, blood values, and lung function analysis. Data were collected by checking electronic medical records, discharge reports and autopsy reports.

The study was approved by the Medical Centre—University of Freiburg’s local ethics committee and conducted in accordance with the guideline proposed in the Declaration of Helsinki. A waiver of consent was granted due to the retrospective nature of the study and the associated minimal risk. It is registered at the German Registry for Clinical Trials (DRKS) under the trial registration number 00021501.

### Follow-up schedule after lung transplantation

In the first year after the lung transplantation, clinical examination, lung function test and surveillance bronchoscopies with bronchoalveolar lavage and lung biopsies are scheduled for 1, 2, 3, 4, 6, and 12 months. From the second postoperative year on, patients are seen every 3 months for clinical examination, lab and lung function testing (Supplemental Fig. 2). In case of conspicuous results, such as infection or an otherwise not explainable decline in lung function, further testing/imaging/bronchoscopy to rule out/confirm CLAD is performed. If clinical symptoms for TE are present, further testing as described under the “definitions” section is initiated.

Immunosuppression medication levels as well as blood and kidney parameters were initially checked weekly after discharge and once stable, the interval was extended to every 4 weeks (Table 1).

**Table 1:** Basic demographic patient characteristics

Variable	All patients (221)
Sex	
Male	115 (52%)
Female	106 (48%)
Age at transplantation	
Minimal age	17 years
Maximal age	69 years
Median age	56 years
< 18 years	1 (0.5%)
Between 18 and 29 years	9 (4%)
Between 30 and 39 years	6 (3%)
Between 40 and 49 years	25 (11%)
Between 50 and 59 years	85 (39%)
≥ 60 years	96 (43%)
BMI at transplantation	
Male	23.9 kg/m^2^
Female	22.5 kg/m^2^
Median	23.2 kg/m^2^
Operation	
Double-lung	196 (89%)
Single-lung	25 (11%)
Underlying disease	
Idiopathic fibrosis	84 (38%)
COPD	77 (35%)
Mucoviscidosis	12 (5%)
Extrinsic allergic alveolitis	12 (5%)
Alpha-1 antitrypsin deficiency	12 (5%)
Other autoimmune disorders	8 (4%)
Sarcoidosis	7 (3%)
LAM	3 (1%)
Re-transplantation	3 (1%)
GvHD	2 (1%)
Other	1 (0.5%)
Cardiovascular diseases	
Arterial hypertension	65 (30%)
Diabetes mellitus	40 (18%)
Coronary heart disease	36 (16%)
Coronary Stent	16 (7%)
Peripheral artery disease	5 (2%)
Hypercholesterolemia	96 (43%)
Atrial fibrillation pre LTX	14 (6%)
Thromboembolism pre LTX	TBA
Cardiovascular therapy pre LTX	
Antiplatelet therapy	31(14%)
Anticoagulants	27(12%)
Statins	47(21%)

Basic patient characteristics before LTX

*COPD* chronic obstructive pulmonary disease, *LAM* Lymphangioleiomyomatosis*, GvHD* Graft-versus-host disease, *ECMO* extracorporeal membrane oxygenation, *BMI* body mass index, *LTX* lung transplantation

### Definitions

We defined TE as the main event. TE includes every event attributed to either thrombotic arterial occlusion (e.g., myocardial infarction, stroke) and venous thrombosis or embolism as listed in Table 2. Thromboembolism was detected mostly by clinical symptoms during regular follow-ups in the transplant outpatient centre. Since asymptomatic patients were not routinely screened, some events might have been missed, especially after the first postoperative year. The diagnosis was established by ultrasound. In case of suspected pulmonary embolism ventilation/perfusion scintigraphy or CT angiography were performed. Diagnostic measures for other venous and arterial events were initiated as appropriate upon clinical presentation. If a patient suffered from multiple thromboembolic events, they were listed separately, each counting as one event. However, for the calculation of survival and risk factors, patients were divided into 2 groups, one with and the other without thromboembolic events. Thromboembolic events that occurred before transplantation were excluded.

**Table 2:** Thromboembolic events

	≤ 1. month	2.–12. months	All events
Thromboembolism	35 (16%)	36 (16%)	113 (51%)
At least one thromboembolic event	26 (12%)	24 (11%)	74 (34%)
More than one thromboembolic event	8 (4%)	10 (5%)	32 (15%)
Venous thromboembolism	**19 (9%)**	**29 (13%)**	**73 (33%)**
Pulmonary embolism	7 (3%)	18 (8%)	34 (15%)
Deep vein thrombosis	3 (1%)	8 (4%)	21 (10%)
Jugular vein thrombosis	7 (3%)	1 (0.5%)	8 (4%)
Thrombi in axillary vein/ subclavian vein	2 (1%)	0 (0.0%)	2 (1%)
CVST	0 (0.0%)	1 (0.5%)	1 (0.5%)
Venous retinal vascular occlusion	0 (0.0%)	0 (0.0%)	1 (0.5%)
TM	0 (0.0%)	1 (0.5%)	4 (2%)
Atrial thrombi	0 (0.0%)	0 (0.0%)	2 (1%)
Arterial thromboembolism	**16 (7%)**	**7 (3%)**	**40 (18%)**
Stroke	13 (6%)	1 (0.5%)	23 (10%)
Abdominal Vascular occlusion	0 (0%)	2 (1%)	5 (2%)
Vascular occlusion in extremities	1 (0.5%)	2 (1%)	7 (3%)
Arterial retinal vascular occlusion	0 (0%)	2 (1%)	2 (1%)
Myocardial infarction	2 (1%)	0 (0%)	3 (1%)

Significance of bold means that p<0.05

Incidence and classification of thromboembolic events

*CVST* Cerebral venous sinus thrombosis, *TM* Thrombotic Microangiopathy

The body mass index (BMI) was used to define if patients are underweight (BMI < 18.5), of normal weight (18.5–24.9), overweight (25.0–29.9) or obese (BMI > 30.0).

### Thrombosis prophylaxis and ICU management

The standard pharmacological thrombosis prophylaxis consisted of 40 mg enoxaparin or 5000 IE unfractionated heparin every 12 h in intensive care unit (ICU) and 4500 IE tinzaparin in intermediate care unit (IMC) every 24 h. This regimen was initiated in ICU as soon as possible after ruling out postoperative active bleeding. Additionally, medical compression bandages were used in ICU and medical compression stockings in IMC and regular wards until discharge. Generally, patients discharged from the hospital did not receive further thrombosis prophylaxis in accordance with the current guidelines. Patients with the indication for therapeutic anticoagulation, e.g., atrial fibrillation or pulmonary embolism, received unfractionated heparin or enoxaparin in therapeutic doses while being at the hospital. Therapeutic anticoagulation therapy was continued after the patients were discharged. After an arterial thromboembolic event patients were placed on aspirin therapy in accordance with current guidelines. Physical therapy was available to all patients starting from the first postoperative day in ICU.

Intravenous as well as intraarterial catheters were removed as soon as possible at the discretion of the treating physician in ICU or IMC.

### Statistical analysis

The Kaplan–Meier-Method was used to estimate overall survival and the log rank test was used for comparison of survival curves of patients with and without TE. To evaluate connections between different parameters the Fischer’s exact test, the Chi-squared test and the Mann–Whitney U test were used when appropriate. Univariate and multivariate logistic regression models were used to select independent predictors of TE and survival in our cohort. All tests were two-tailed. A p-value < 0.05 was considered statistically significant. All statistical analyses were conducted using SPSS software (Version 27, IBM Corporation, New York, NY, USA) and GraphPad Prism (Version 9, GraphPad Software, San Diego, CA 92108, USA).

#### Results

**Fig. 1:**
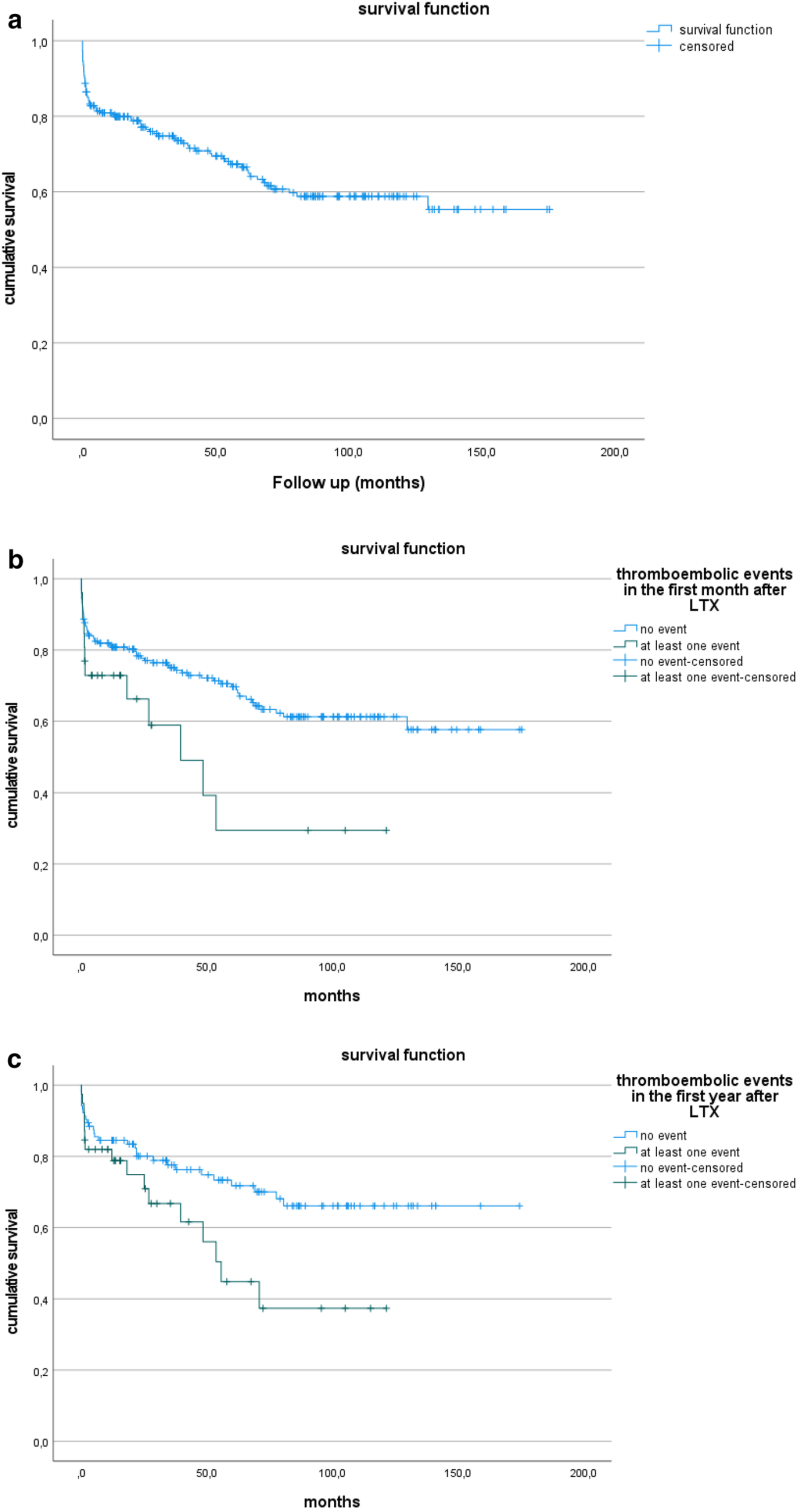
**a** Overall post-transplant survival. Kaplan–Meier Analysis of survival after lung tranplantation. 1 month: 89%, 6 months: 82%, 1 year: 80%, 5 years: 66% and10 years 59%. **b** overall survival and survival after thromboembolic events within the first month after LTX. Log rank t test p = 0.027. Survival after lung tranplantation. Thromboembolic event in the first month (1 year: 74%, 3 years: 60%, 5 years: 30% and 10 years: 30%) vs. no thromboembolic event in the first month (1 year: 81%, 3 years: 75%, 5 years: 70% and 10 years: 61%). **c** overall survival and survival after thromboembolic events within the first year after LTX of patients ≥ 55 years. Long rank t test p = 0.028. Survival after lung tranplantation. Thromboembolic event in the first year (1 year: 82%, 3 years: 67%, 5 years: 45% and 10 years: 37%) vs. no thromboembolic event in the first year (1 year: 85%, 3 years: 78%, 5 years: 72% and 10 years: 66%) in patients ≥ 55 years of age

Overall, 221 patients (115 male, 106 female) underwent LTX at our institution between March 2002 and June 2021. 196 (89%) patients underwent a double lung transplantation, 25 (11%) patients a single lung transplantation. 68 (31%) patients underwent LTX before implementation of the lung allocation score (LAS)—based distribution system at the end of 2011. From 2012 until 2021, another 153 transplantations (69%) were performed. At the time of data analysis, 146 patients (66) were still alive. The 1-year-, 5-years- and 10-years-survival-rate in our patient collective is 80%, 66% and 59% respectively (Fig. 1a).

The average waiting time for a transplantation was 14 months (range 2 days to 11 years). The main underlying pulmonary diseases leading to LTX were idiopathic fibrosis (38%), or chronic obstructive pulmonary disease (COPD) (35%). Concomitant cardiovascular diseases, e.g., arterial hypertension (29%) or one-vessel coronary artery disease (17%), as well as diabetes mellitus (18%), were present in a substantial fraction of the patients (Table 3). Patients transplanted after 2011 were more often aged > 55 years compared to patients that underwent surgery before the implementation of the LAS score (72% vs. 49%, p < 0.001). Overall, 143 patients (65%) were over 55 years old, and 96 patients (43%) were aged 60 years or older at the time of transplantation (Table 1).

**Table 3:** Factors associated with thromboembolism (TE)

Variable	All patients (n = 221)	Patients without TE (n = 147)	Patients with TE (overall) (n = 74)	p	Venous TE (all events) (n = 73)	p	Arterial TE (all events) (n = 40)	p
Primary lung disease								
Idiopathic fibrosis	84 (38%)	58 (40%)	26 (35%)	0.560	21(28%)	0.513	8(20%)	0.117
COPD	77 (35%)	45 (31%)	32 (43%)	0.073	19(26%)	0.132	16(40%)	0.070
Mucoviscidosis	12 (5%)	10 (7%)	2 (3%)	0.345	1(1%)	0.306	1(3%)	1.000
EAA	12 (5%)	8 (5%)	4 (5%)	1.000	2(3%)	1.000	1(3%)	1.000
Alpha-1 antitrypsin deficiency	12 (5%)	10 (7%)	2 (3%)	0.345	1(1%)	0.306	1(3%)	1.000
Sarcoidosis	7 (%)	4 (3%)	3 (4%)	0.689	1 (1%)	1.000	1(3%)	1.000
Operation								
Double-lung	196 (88.7%)	133 (91%)	63 (85%)	0.264	41(56%)	0.125	29(73%)	1.000
Single-lung	25 (11.3%)	14 (10%)	11 (15%)	0.264	9(12%)	0.125	3(8%)	1.000
ECMO	78 (35.3%)	59 (40%)	19 (26%)	**0.037**	15(21%)	0.405	9(23%)	0.427
Age								
≥ 55 years	143 (65%)	88 (60%)	55 (74%)	**0.037**	40(55%)	**0.011**	23(58%)	0.427
≥ 60 years	96 (43%)	56 (38%)	40 (54%)	**0.031**	30(41%)	**0.009**	18(45%)	0.126
BMI*								
< 18,5 kg/m^2^	33 (15%)	28 (19%)	5 (7%)	**0.016**	0(0%)	** < 0.001**	5(13%)	1.000
18,5–24,9 kg/m^2^	111 (50%)	75 (51%)	36 (49%)	0.776	22(30%)	0.336	17(43%)	0.849
≥ 25 kg/m^2^	76 (34%)	43 (30%)	33 (45%)	**0.035**	28(38%)	** < 0.001**	10(25%)	0.841
≥ 30 kg/m^2^	11 (5%)	2 (1%)	9 (12%)	**0.001**	7(10%)	**0.003**	2(5%)	0.661
Cardiovascular diseases								
Arterial hypertension	65 (29%)	34 (23%)	31 (42%)	**0.005**	24(33%)	**0.003**	9(23%)	1.000
Diabetes mellitus	40 (18%)	19 (13%)	21 (28%)	**0.009**	16(22%)	**0.006**	9(23%)	0.136
Coronary heart disease	36	22 (15%)	14 (19%)	0.427	10(14%)	0.499	6(15%)	0.595
PAVK	5 (2%)	1 (1%)	4 (5%)	**0.043**	3(4%)	0.075	4(10%)	** < 0.001**
Hypercholesterolemia	96 (43%	66 (45%)	30 (41%)	0.452	21(29%)	0.737	11(28%)	0.230
Atrial fibrillation pre LTX	14 (6%)	13 (9%)	1 (1%)	**0.039**	1(1%)	0.202	1(3%)	0.698
Atrial fibrillation post LTX	66 (30%)	47(31%)	19(26%)	0.353	11(15%)	0.219	9(23%)	1.000

Significance of bold means that p<0.05

Patient characteristics and concomitant diseases in patients with and without thromboembolism

*COPD* chronic obstructive pulmonary disease, *ECMO* extracorporeal membrane oxygenation, *BMI body mass index, LTX* lung transplantation

^*^The BMI at the time of transplant is unknown for one patient (external LTX)

We identified 74 patients (33%) who experienced at least one thromboembolic event after lung transplantation. Most of the events (68%) took place within the first postoperative year, whereas 35% already occurred within the first postoperative month (Table 1). 34 patients (15%) developed pulmonary embolism and 23 patients (10%) were diagnosed with ischemic stroke. Hemiparesis or hypaesthesia persisted in 11 patients (4.8%) of the latter group. Most pulmonary embolisms occurred during the first year (53%) while the majority of strokes took place in the first month (57%). However, approximately 1/3 of all thromboembolic events were diagnosed after the first postoperative year (Table 1).

Thromboembolic events within the first postoperative month were associated with reduced survival after transplantation (56% vs. 68%, p = 0.027) (Fig. 1b). Notably, patients over 55 years of age at the time of transplantation, which comprise 65% of our cohort, had not only an increased incidence of thromboembolic events in the first postoperative month (p = 0,025), but also a reduced survival rate when they experienced at least one event within the first year (59% vs. 72%, p = 0,028) (Fig. 1c). In accordance with that, a recipient age of > 55 years correlated with the incidence of TE (p = 0.037) (Table 3). Moreover, arterial hypertension (p = 0.004), peripheral artery disease (p = 0.019) and diabetes mellitus (p = 0.017) were independent predictors for TE (Table 4). These factors are closely related to the metabolic syndrome, and fittingly, a body mass index (BMI) of > 25 at the time of transplantation also contributed significantly to the risk for TE (p = 0.035) (Tables 3 and 4). Particularly venous TE correlated with the above-mentioned factors with few exceptions when arterial TE was more predominant (Tables 3 and 4). While several independent risk factors for TE could be identified, they had no effect on long-term survival (Table 5).

**Table 4:** Logistic regression analysis of factors associated with TE

Variables	Wald	Exp(B)	95%CI Exp(B)		p
Overall			Min.	Max.	
Peripheral artery disease	2.313	10.103	1.044	97.738	**0.046**
BMI ≥ 30 kg/m^2^	7.41	9.215	1.862	45.598	**0.006**
Diabetes mellitus	4.315	2.213	1.046	4.681	**0.038**
Hypertension	7.289	2.412	1.273	4.571	**0.007**
Venous TE					
Peripheral artery disease	3.816	6.882	0.994	47.666	0.051
BMI ≥ 30 kg/m^2^	7.064	6.186	1.614	23.715	**0.008**
Diabetes mellitus	4.461	2.356	1.064	5.218	0.035
Hypertension	9.31	2.987	1.497	6.032	**0.002**
Arterial TE					
Peripheral artery disease	8.509	27.556	2.968	255.795	**0.004**

Significance of bold means that p<0.05

Forward stepwise logistic regression analysis of factors associated with TE

*BMI* body mass index

**Table 5:** Univariate and multivariate Cox regression analyses to identify predictors of survival

Variables (n = 221)	Univariate, HR (95% CI)	p	Multivariate, HR (95% CI)	p
Age				
< 55 years	1.270 (0.800–2.017)	0.311		
≥ 55 years	0.787 (0.496–1.251)	0.311		
Sex				
Gender (female)	1.825 (1.150–2.897)	**0.011**	1.898 (1.193–3.019)	**0.007**
Underlying lung disease				
Fibrosis	1.441 (0.914–2.271)	0.116		
COPD	0.530 (0.315–0.893)	**0.017**	0.574 (0.329–1.002)	0.051
Operation				
Double lung	1.033 (0.515–2.075)	0.927		
Single lung	0.968 (0.482–1.944)	0.927		
ECMO	1.901 (1.207–2.993)	**0.006**	1.579 (0.969–2.573)	0.067
BMI				
< 18.5 kg/m^2^	1.313 (0.733–2.351)	0.360		
≥ 25 kg/m^2^	1.191 (0.737–1.924)	0.475		
Concomitant diseases				
PAD	2.221 (0.698–7.065)	0.177		
Diabetes mellitus	1.306 (0.741–2.304)	0.356		
CHD	0.813 (0.409–1.613)	0.553		
Arterial hypertension	0.869 (0.520–1.450)	0.591		
Hypercholesterolemia	0.974 (0.599–1.586)	0.917		
Autoimmune disease	1.506 (0.828–2.742)	0.180		
Atrial fibrillation	0.516 (0.162–1.638)	0.261		
mPAP ≥ 25 mmHG	0.923 (0.550–1.550)	0.763		
Thromboembolism				
≤ 30 days	1.994 (1.067–3.728)	**0.031**	1.860 (0.985–3.511)	0.056
≤ 1 year	1.292 (0.747–2.235)	0.359		
All events	1.030 (0.640–1.657)	0.904		
Pulmonary embolism				
≤ 30 days	1.815 (0.568–5.804)	0.315		
≤ 1 year	0.633 (0.255–1.574)	0.326		
All events	0.538 (0.247–1.172)	0.119		
Stroke				
≤ 30 days	1.453 (0.584–3.612)	0.422		
≤ 1 year	1.336 (0.537–3.324)	0.533		
All events	1.343 (0.689–2.616)	0.387		

Significance of bold means that p<0.05

Predictors of survival

*COPD* chronic obstructive pulmonary disease, *ECMO* extracorporeal membrane oxygenation, *BMI* body mass index, *PAD* Peripheral artery disease, *CHD* coronary heart disease, *mPAP* mean pulmonary artery pressure

At the time of transplantation, about half of patients (51%) had a normal weight with a BMI between 18.5 and 24.9 kg/m^2^ (Supplementary Fig. 1). In our cohort, 36 (32%) patients of normal weight had a thromboembolic event and 42% of them were diagnosed with pulmonary embolism. The incidence of pulmonary embolism further increased to 61% in patients with a BMI of > 25. In contrast, only 5 (15%) underweight patients were diagnosed with TE and none of them had pulmonary embolism. This indicates that specifically the occurrence of pulmonary embolism was closely related to the patients’ weight (p < 0.001).

## Discussion

### Incidence and timing of TE

We found a 30-day and 12-month incidence of TE of 12% and 23% respectively in our lung transplant recipients. Overall, about one third of them had at least one thromboembolic event during the postoperative course. Current evidence indicates that the incidence of venous TE after LTX is higher [6] compared to other cardiothoracic surgeries, but varies between studies (8%–43%) [6, 12, 13, 24, 25]. A possible explanation is that the lung transplant cohorts are heterogeneous due to different underlying diseases and preexisting conditions. Moreover, there are also differences in screening protocols and thrombosis prophylaxis regimes as well as in time schedules of follow-up appointments between institutions. Notably, many cases of TE occur during hospital stay despite the use of thrombosis prophylaxis. It is widely accepted that pharmacologic prophylaxis with unfractionated heparin and low molecular weight heparin should be monitored with appropriate tests such as anti-factor-Xa-activity, for example. This may help to tailor individual doses for every patient. Although most thromboembolic events in our cohort took place in the first postoperative year, about one third was detected later than that. It can only be assumed that with a structured screening in place, the incidence would be even higher. In accordance with this, a high risk for recurrence was shown in a study by Prandoni et al., who followed a cohort of 1626 consecutive patients with venous TE in Padua, Italy for up to 10 years and found a high rate of recurrent events: 11% after 1 year, 20% after 3 years, 29% after 5 years, and 40% after 10 years [26].

### Risk factors and distribution of TE

We identified both, arterial and venous thromboembolic events. Both share common risk factors [27], which can be found in an increasing fraction for TE in our cohort. Although they share similar risk factors, they are different diseases and, in our cohort, venous events are more common than the arterial ones. Interestingly, most of the risk factors seem to be statistically relevant for venous TE which makes a prolonged thrombosis prophylaxis even more relevant.

We had a surprisingly high number of patients suffering from stroke, not only in the first month after surgery but also after the first postoperative year. Transplantation surgery itself along with the above-mentioned risk factors have been described to play a role in the early cases of those occurring within 30 days [28]. The later ones, however, may be a combined result of preexisting condition and e.g. the immunosuppression or other factors in the aftermath of the transplantation. Patients with newly diagnosed atrial fibrillation after surgery were placed on anticoagulation therapy according to current guidelines, and there is no statistically significant increase in the incidence of stroke in these patients (Table 3). We may, however, have missed some cases of atrial fibrillation if it occurred later or only paroxysmal and without symptoms.

Perioperative ECMO support itself did not increase the risk for TE in our cohort, however, this may be due to the large time span of observation and the fact that 30-days-mortality in patients on ECMO support as ‘bridge to transplant’ was much higher in the earlier years compared to the last decade.

There are some reports describing thrombi arising from the surgical suture lines, which is a possible, although rare source for TE [29, 30]. The venous and pulmonary arterial anastomoses are checked for patency and flow pattern intra- and, if needed, also postoperatively by transesophageal echography. Moreover, a precise suture technique with an endothelium-to-endothelium junction to occlude the muscle from the blood-contacting surfaces is employed for the venous cuff anastomoses [29]. All patients are checked for an atrial septum defect before transplantation and occlusion therapy is initiated for patients at need.

As described in previous studies, weight plays an important role in the incidence of thromboembolic events [31–33]. In our cohort, almost half of the patients with a BMI > 25 had a thromboembolic event during the postoperative course, most of them within the first year after transplantation. In recent years, our patients who initially presented with a BMI > 30 had to reduce weight before transplantation as this reduces not only the risk for TE and other cardiovascular complications but also for the development of primary graft dysfunction [32].

### Prophylaxis and treatment of venous and arterial TE

All patients at our center received thrombosis prophylaxis and regular physiotherapy with the goal of ambulation starting from ICU until the day of discharge. Furthermore, all patients with TE were prescribed anticoagulation therapy or antiplatelet drugs according to current guidelines. Pre-existing antiplatelet therapy was not interrupted for transplantation and pre-existing anticoagulation therapy was continued as soon as possible after surgery with low molecular weight heparin in therapeutic doses. Patients with atrial fibrillation known prior to LTX were on anticoagulation therapy and none of them suffered postoperative stroke.

Gastrointestinal bleeding occurred in 6 (3%) patients on anticoagulation after transplantation, while no other major bleeding events were recorded. However, individual risks for bleeding complications need to be taken into consideration when thinking about a prolonged thrombosis prophylaxis for a certain amount of time to reduce the incidence of TE for patients at risk.

### Limitations of the study

A limiting factor of this retrospective single-centre study is that it covers a period of nearly 20 years and thus our findings may not apply to all lung transplant recipients. Moreover, due to the absence of clinical signs, we might have missed some cases of TE or complications thereof in our patient cohort. This is especially true for events that occurred after the first postoperative year. That said, a strength of the study is that one single protocol regarding postoperative management and TE prophylaxis applied to all patients at the respective time of transplantation, despite the arguably long follow-up time. The latter, however, allowed us to detect the events that occurred years after transplantation.

#### Conclusions

About 2/3 of all TE events occur in the first postoperative year, therefore an extended thrombosis prophylaxis for lung transplant patients with risk factors for TE such as age of more than 55 years, cardiovascular risk factors or diabetes mellitus, seems beneficial, particularly within the first year after surgery. However, whether this reduces the incidence of TE, or whether therapeutic anticoagulation is beneficial for selected patients for a certain amount of time after LTX needs to be analysed in randomised controlled studies. Considering the individual risk of the patient, also with regards to potential bleeding complications, is essential when making a decision.

In any case, it is very important to be aware of the increased risk of TE and to improve early detection, particularly in patients with pre-existing or new-onset cardiovascular comorbidity. The implementation of a regular screening, possibly integrated in the follow-up schedule at the outpatient transplant centre, as described for example by Zheng et al. [12] or Jorge et al [34], seems to be of great value and should be subject to further investigation.

## Supplementary Information

Below is the link to the electronic supplementary material.

Supplementary file1 (SVG 71 KB)—Supplementary Fig. 1 Distribution of patients’ weight at the time of lung transplantation

Supplementary file2 (PNG 214 KB)—Supplementary Fig. 2 Follow-up schedule after lung transplantation

## Data Availability

The dataset generated during and/or analysed during the current study is available from the corresponding author on reasonable request.
