# Supplementary figures and images for: Independent risk factors for an increased incidence of thromboembolism after lung transplantation
Source: J Thromb Thrombolysis. 2022 Dec 10;55(2):252–62. doi: 10.1007/s11239-022-02748-9 (PMC10011327; doi:10.1007/s11239-022-02748-9)

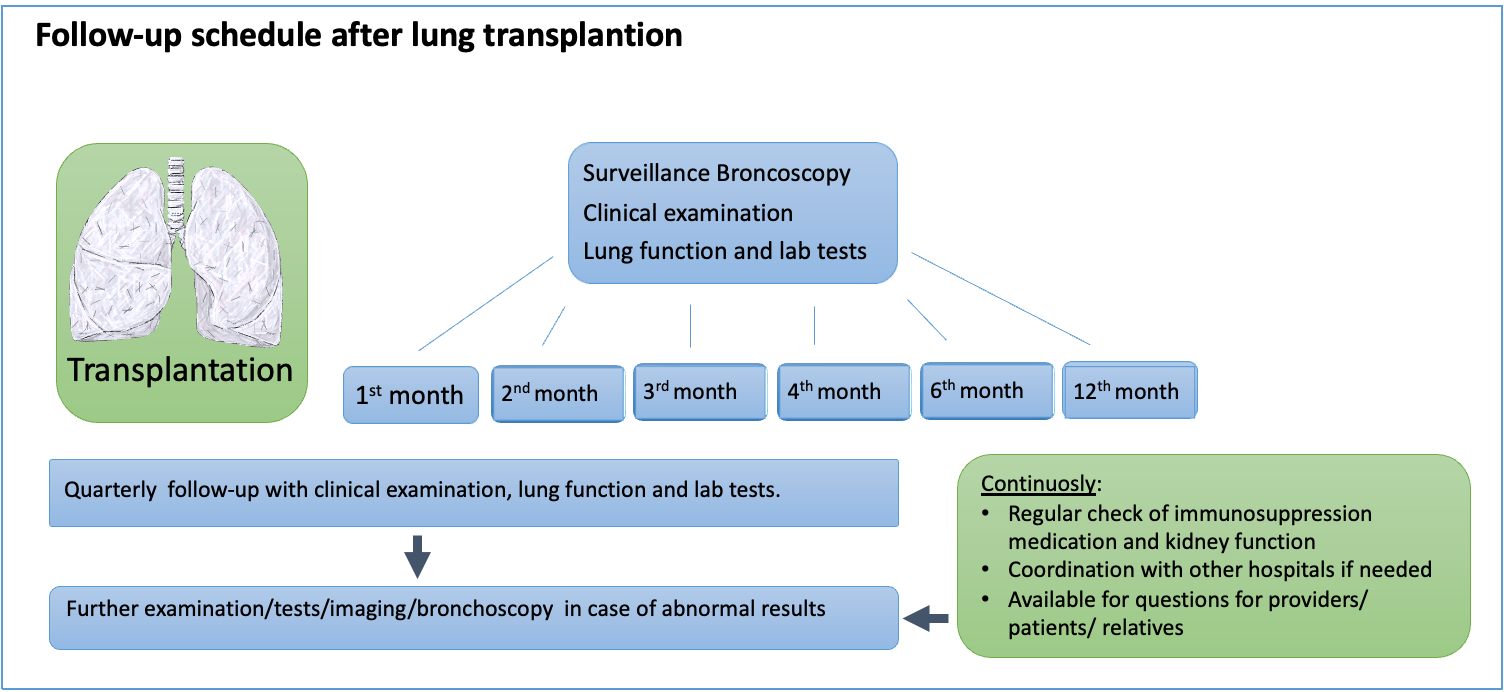

Supplement: Supplementary file 2 — Supplementary file2 (PNG 214 KB)—Supplementary Fig. 2 Follow-up schedule after lung transplantation [file 11239_2022_2748_MOESM2_ESM.png]
